# Des localisations inhabituelles des lipomes sous cutanés: rapport de cas

**DOI:** 10.11604/pamj.2022.42.300.28318

**Published:** 2022-08-22

**Authors:** Yassamina Ribag, Abdelhafid Achbouk, Jalal Hamama, Mohamed Karim EL Khatib

**Affiliations:** 1 Service de Chirurgie Plastique et de Stomatologie, Faculté de Médecine et de Pharmacie, Hôpital Militaire d’Instruction Mohammed V, Rabat, Maroc

**Keywords:** Lipome, localisations inhabituelles, cas clinique, Lipoma, unusual locations, case report

## Abstract

Les lipomes sous-cutanés sont des tumeurs, très fréquentes qui siègent préférentiellement au niveau du cou et du tronc. Cependant, d´autres localisations peuvent se voir, bien qu´elles soient rares et inhabituelles, et le médecin doit penser au lipome, devant toutes tuméfactions sous cutanées y siégeant. Les auteurs rapportent 3 cas de localisations rares des lipomes, tout en détaillant le diagnostic et le traitement dont ces patients ont bénéficié. Ces localisations sont la première commissure interdigitale de la main, le doigt de pied et le canthus externe de l´œil.

## Abstract (en)

Subcutaneous lipomas are very common tumors preferentially occurring in the neck and trunk. Although rare and unusual, they can occur in other parts of the body. Then they should be suspected in patients with subcutaneous swellings. We here report 3 cases of lipomas occurring in rare sites, detailing diagnosis and treatment. They occurred in the interdigital web space of the hand, toe and lateral canthus of the eye.

## Introduction

Les lipomes sous-cutanés sont des tumeurs mésenchymateuses bénignes qui se développent dans le tissu adipeux. Ils sont très fréquents, puisqu´ils représentent près de 50% de toutes les tumeurs des tissus mous [1]. Ils apparaissent principalement chez les adultes âgés de 40 à 60 ans, sans prédominance de sexe. Ils peuvent être post-traumatiques [2]. Toutes les régions graisseuses du corps, peuvent être le siège de ces tumeurs [2], cependant certaines localisations telles que la région intra-orbitaire, le pied et la main restent rares [3]. A travers ce travail, nous rapportant 3 cas cliniques de patients présentant des lipomes dont la localisation est inhabituelle, diagnostiqués dans le service de chirurgie plastique et maxillo-faciale de l´hôpital militaire d´instruction Mohamed V de Rabat au Maroc, sur une période de 5 ans, de janvier 2015 à décembre 2020.

## Patient et observation

### Cas clinique 1

**Information de la patiente:** patiente de 39 ans, sans antécédents pathologiques. Elle consulte pour une tuméfaction du canthus externe de l´œil gauche.

**Résultats cliniques:** l´examen clinique retrouve une masse siégeant au niveau du canthus externe de l´œil gauche et couvrant latéralement et partiellement le côté gauche de la cornée.

**Figure 1:**
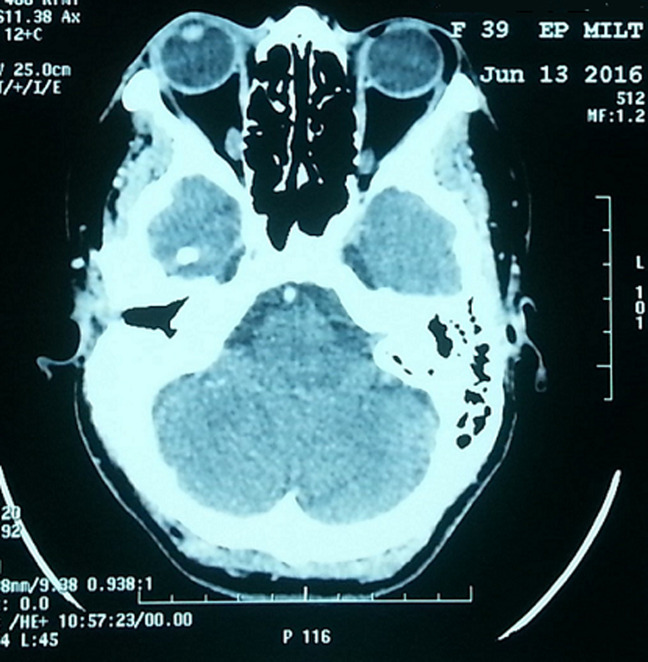
image radiologique d´une tomodensitométrie (TDM) objectivant un lipome du canthus supéro-externe de l´orbite gauche

**Démarche diagnostique:** une tomodensitométrie (TDM) réalisée a objectivé la présence au niveau du canthus supéro-externe de l´orbite gauche d´un lipome mesurant 13.5x5.5 mm (Figure 1).

**Figure 2:**
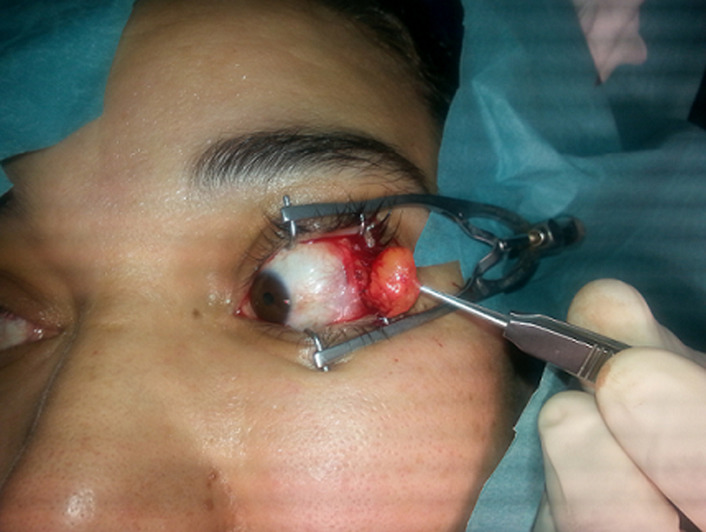
ablation du lipome intra-orbitaire, image per-opératoire

**Intervention thérapeutique et suivi:** l´ablation du lipome a été réalisée sous anesthésie locale (Figure 2). La nature lipomatose de la tumeur a été confirmée par l´examen anatomopathologique.

### Cas clinique 2

**Information du patient:** patient de 55 ans, tabagique chronique, consulte pour une tuméfaction de la première commissure interdigitale de la main droite.

**Figure 3:**
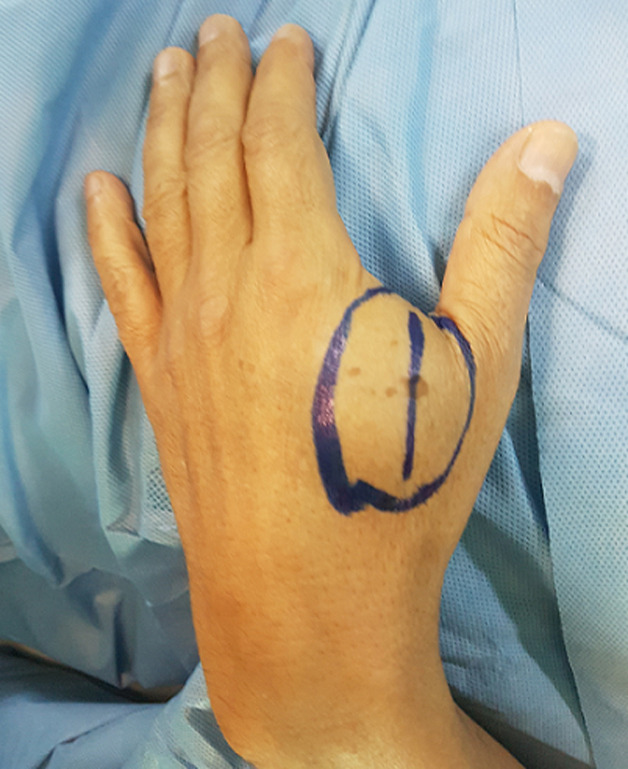
tuméfaction de la face dorsale de la main droite

**Résultats cliniques:** l´examen clinique retrouve une masse siégeant au niveau de la première commissure interdigitale de la main droite. Elle est ovalaire, molle mobile, indolore et mesurant environ 5 cm de grand axe (Figure 3).

**Figure 4:**
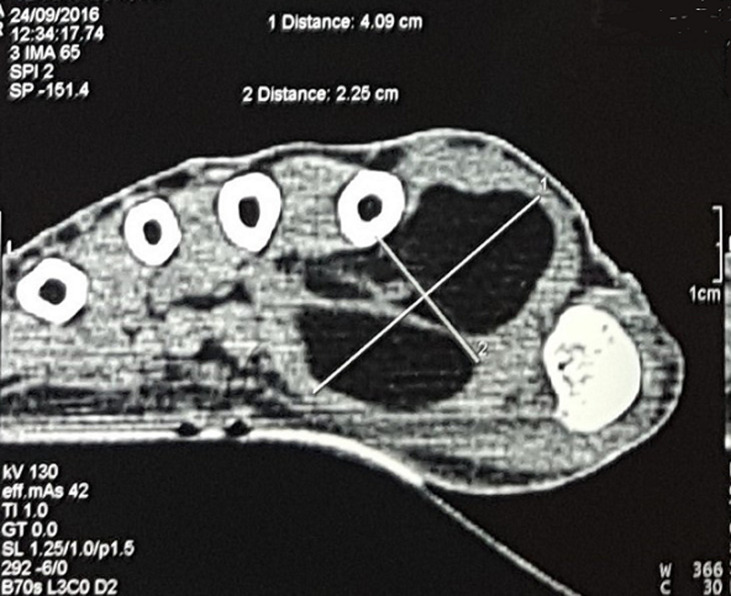
image radiologique tomodensitométrie (TDM) d´une formation d´allure lipomateuse au niveau du premier espace interdigital de la main droite

**Démarche diagnostique:** une TDM réalisée a objectivé la présence au niveau de la commissure interdigitale de la main droite, d´une formation d´allure lipomatose mesurant 40x25 mm (Figure 4).

**Figure 5:**
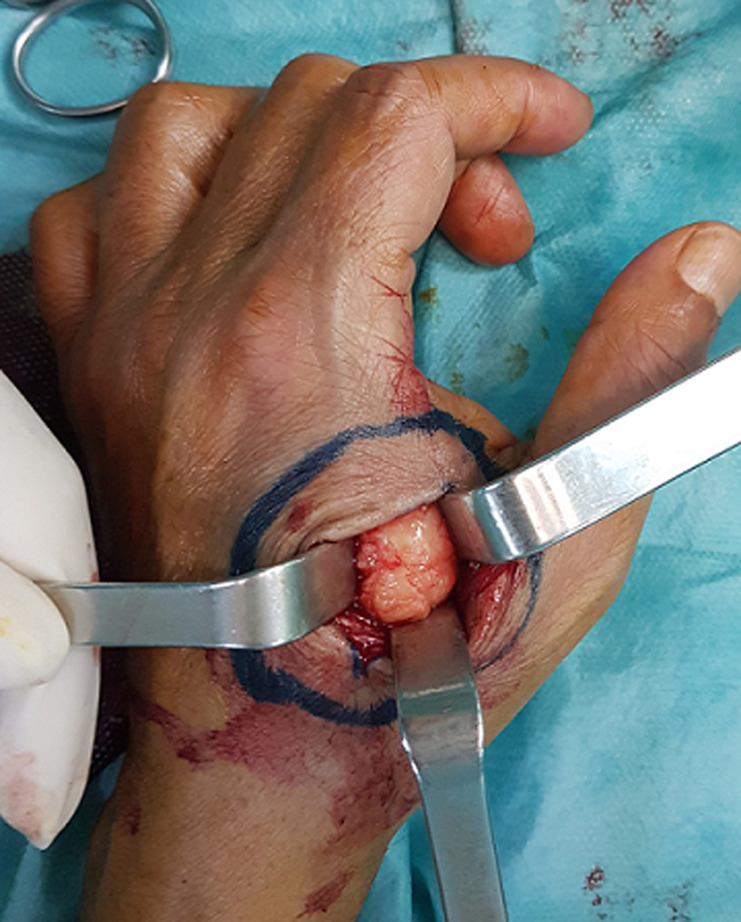
ablation d´un lipome de la première commissure interdigitale de la main droite, per-opératoire

**Intervention thérapeutique et suivi:** le patient a bénéficié de l´ablation du lipome sous anesthésie locorégionale (Figure 5). La nature lipomatose de la tumeur a été confirmée par l´examen anatomopathologique.

**Table 1:** les données cliniques, paracliniques et thérapeutiques des patients

Cas clinique	Sexe	Age	Taille	Localisation	Radiologie	Traitement
1	F	39 ans	13.5/5.5 mm	Canthus supéro externe de l'orbite gauche	TDM: lipome du canthus supéro externe de l'orbite gauche	Exérése chirurgicale sous Al
2	H	55 ans	40/25 mm	1^ére^ commissure interdigitale de la main droite	TDM: formation d'allure lipomateuse	Exérése chirurgicale sous Al
3	H	73 ans	23/13 mm	2^e^ doigt du pied gauche	Echographie: image d'échostructure tissulaire, au contact du 2^e^ métatarse	Exérése chirurgicale sous Al

### Cas clinique 3

**Information du patient:** un homme de 73 ans, diabétique sous antidiabétiques oraux, consulte pour une tuméfaction du deuxième doigt du pied gauche, augmentant progressivement de taille et gênant le port de chaussure.

**Figure 6:**
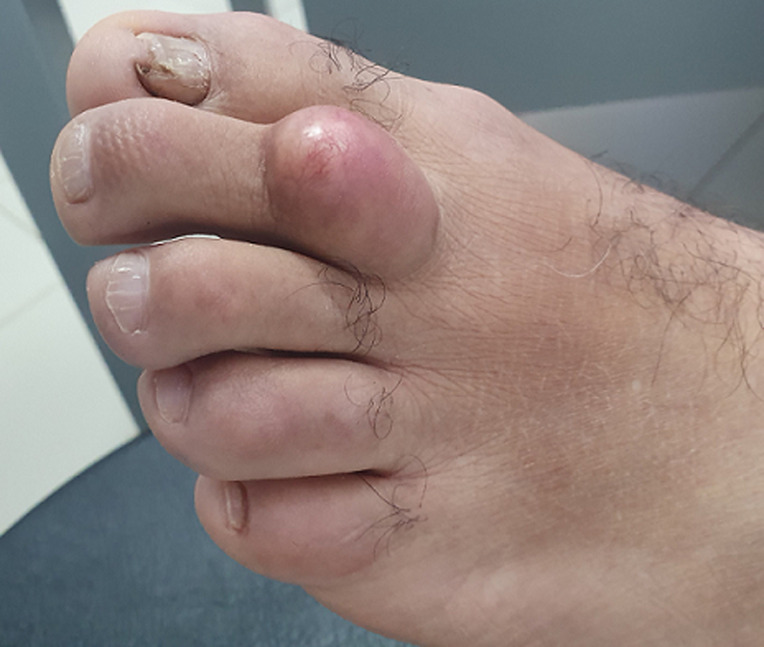
tuméfaction en regard du deuxième orteil du pied gauche correspondant à un lipome sous cutané

**Résultats cliniques:** l´examen clinique retrouve une masse siégeant sur la face dorsale du deuxième doigt du pied gauche. Elle est ovalaire, mobile, non adhérente au plan profond, indolore et mesurant environ 2.5 cm de diamètre (Figure 6). Démarche diagnostique: une échographie réalisée a objectivé la présence d´une image d´échostructure tissulaire, au contact du 2^e^ métatarse du pied gauche et mesurant 23x13 mm.

**Intervention thérapeutique et suivi:** le patient a bénéficié de l´ablation du lipome sous anesthésie locale. La nature lipomatose de la tumeur a été confirmée par l´examen anatomopathologique. Au jour d´aujourd´hui, et chez les 3 patients, aucune récidive n´a été rapportée.

## Discussion

Les lipomes sont des tumeurs mésenchymateuses bénignes des plus fréquentes. Ils sont constitués de cellules graisseuses matures pouvant être entourées ou non d´une capsule de tissu conjonctif. Elles se développent dans toutes les régions graisseuses du corps [2]. Leurs sièges de prédilection sont le tronc, le cou et l´extrémité proximale des membres, cependant, et pour des raisons que nous ignorons, leur localisation sur les mains, les pieds et Le premier cas de lipome de la main a été décrit par McEnery [4] en 1959. Généralement, ils siègent au niveau de la paume et de la face palmaire des doigts [4]. Les lipomes du pied sont tout aussi rares et représentent environ 3 à 4% des tumeurs du pied [5,6]. Ils se situent principalement au niveau du dos du pied et de la cheville [5]. Leur localisation au niveau de la plante du pied est encore plus rare car le tissu adipeux y est rare et peut être handicapante. Les lipomes sont exceptionnellement retrouvés au niveau des doigts, leur incidence est de 1%. Le premier cas de lipome du doigt a été publié en 1959 par Stein [7]. Les lipomes intra orbitaires, sont exceptionnels, car les tumeurs mésenchymateuses intra-orbitaire, ne représentent que 5% des tumeurs de l´orbite [3]. Les lipomes cutanés se présentent cliniquement sous la forme d´une masse sous-cutanée ferme, élastique, compressible, mobile par rapport au plan profond, généralement indolore et de taille progressivement croissante [2] la peau en regard est généralement d´apparence normale. Quand ils siègent au niveau de la main, ces tumeurs se manifestent souvent par un syndrome compressif, surtout neurologique [8] dont la symptomatologie clinique diffère selon le siège du lipome. Au niveau des pieds, ils siègent en profondeur entre les gaines tendineuses et les loges musculaires [6] et surviennent généralement après un traumatisme, soit suite à une hernie du tissu adipeux par une ouverture dans l'aponévrose, soit suite à la libération de cytokines qui stimulent la différenciation des préadipocytes en adipocytes matures [5]. Ces lipomes sont caractérisés par leur évolution lente et asymptomatique, qui retarde souvent leur diagnostic. L´étiologie et la pathogenèse du lipome ne sont toujours pas claires. Des facteurs génétiques, traumatiques et métaboliques ont été évoqués [9]. Le diagnostic positif des lipomes repose généralement sur la clinique et l´imagerie radiologique: l´échographie, la TDM ou l´ Imagerie par résonance magnétique (IRM) qui reste la technique de choix pour l´investigation des tumeurs des tissus mous du pied, elle donne une excellente résolution anatomique ainsi qu´un bon contraste des tissus mous, elle pose souvent le diagnostic avec spécificité [10,11]. L´exérèse chirurgicale reste le traitement de choix et l´examen histologique confirme la nature adipeuse de la tumeur.

## Conclusion

Le diagnostic de lipome doit être évoqué devant toute tuméfaction se développant dans un tissu graisseux, quel que soit son siège.
